# Association of clinical, laboratory and imaging biomarkers with the occurrence of acute myocardial infarction in patients without standard modifiable risk factors – rationale and design of the “Beyond-SMuRFs Study”

**DOI:** 10.1186/s12872-023-03180-4

**Published:** 2023-03-23

**Authors:** Dimitrios V. Moysidis, Stylianos Daios, Vasileios Anastasiou, Alexandros C. Liatsos, Andreas S. Papazoglou, Efstratios Karagiannidis, Vasileios Kamperidis, Kali Makedou, Aikaterini Thisiadou, Paraskevi Karalazou, Marios Papadakis, Christos Savopoulos, Antonios Ziakas, George Giannakoulas, Vassilios Vassilikos, Georgios Giannopoulos

**Affiliations:** 1 Third Department of Cardiology, Hippokration General Hospital, School of Medicine, Faculty of Health Sciences, Aristotle University of Thessaloniki, Konstantinoupoleos 49, Thessaloniki, 54642 Greece; 2 First Department of Cardiology, School of Medicine, Faculty of Health Sciences, AHEPA University Hospital, Aristotle University of Thessaloniki, St. Kyriakidi 1, 54636 Thessaloniki, Greece; 3 Athens Naval Hospital, Dinokratous 70, Athens, 11521 Greece; 4 Laboratory of Biochemistry, Faculty of Health Sciences, School of Medicine, AHEPA General Hospital, Aristotle University of Thessaloniki, St. Kyriakidi 1, Thessaloniki, 54636 Greece; 5 University Hospital Witten-Herdecke, University of Witten-Herdecke, Heusnerstrasse 40, 42283 Wuppertal, Germany; 6 First Propedeutic Department of Internal Medicine, Aristotle University of Thessaloniki, AHEPA University Hospital of Thessaloniki, Thessaloniki, Greece

**Keywords:** Coronary artery disease, Acute coronary syndrome, Standard modifiable risk factors, SmuRFs, Predictive biomarkers, Personalized medicine

## Abstract

**Background:**

Acute myocardial infarction (AMI) remains the leading cause of mortality worldwide. The majority of patients who suffer an AMI have a history of at least one of the standard modifiable risk factors (SMuRFs): smoking, hypertension, dyslipidemia, and diabetes mellitus. However, emerging scientific evidence recognizes a clinically significant and increasing proportion of patients presenting with AMI without any SMuRF (SMuRF-less patients). To date, there are no adequate data to define specific risk factors or biomarkers associated with the development of AMIs in these patients.

**Methods:**

The ‘‘Beyond-SMuRFs Study’’ is a prospective, non-interventional cohort trial designed to enroll patients with AMI and no previous coronary intervention history, who undergo coronary angiography in two academic hospitals in Thessaloniki, Greece. The rationale of the study is to investigate potential relations between SMuRF-less AMIs and the clinical, laboratory and imaging profile of patients, by comparing parameters between patients with and without SMuRFs. Complete demographic and comprehensive clinical data will be recorded, Venous blood samples will be collected before coronary angiography and the following parameters will be measured: total blood count, standard biochemistry parameters, coagulation tests, hormone levels, glycosylated hemoglobin, N- terminal pro-B-type natriuretic peptide and high-sensitivity troponin T levels- as well as serum levels of novel atherosclerosis indicators and pro-inflammatory biomarkers. Furthermore, all participants will undergo a complete and comprehensive transthoracic echocardiographic assessment according to a pre-specified protocol within 24 h from admission. Among others, 2D-speckle-tracking echocardiographic analysis of cardiac chambers and non-invasive calculation of myocardial work indices for the left ventricle will be performed. Moreover, all patients will be assessed for angiographic parameters and the complexity of coronary artery disease using the SYNTAX score. Multivariable linear and logistic regression models will be used to phenotypically characterize SMuRF-less patients and investigate independent clinical, laboratory, echocardiographic and angiographic biomarkers-predictors of SMuRF-less status in AMI.The first patient was enrolled in March 2022 and completion of enrollment is expected until December 2023.

**Discussion:**

The ‘‘Beyond-SmuRFs’’ study is an ongoing prospective trial aiming to investigate potential clinical, laboratory and imaging biomarkers associated with the occurrence of AMIs in SMuRF-less patients. The configuration of these patients’ profiles could lead to the development of personalized risk-stratification models predicting the occurrence of cardiovascular events in SΜuRF-less individuals.

**Trial Registration:**

ClinicalTrials.gov Identifier: NCT05535582 / September 10, 2022.

## Background

Acute myocardial infarction (AMI) remains the leading cause of mortality worldwide [1]. The incidence of coronary artery disease (CAD) and -its most adverse manifestation- AMI, has been proven to rise along with the increasing prevalence of major cardiovascular risk factors, such as obesity, smoking, and hypercholesterolemia [2]. These comorbidities have been well recognized as risk factors of coronary artery disease (CAD) and are often used to evaluate the risk of sustaining an acute coronary event, including AMI. Furthermore, the primary and secondary prevention of AMIs has primarily focused on the modification and treatment of standard modifiable risk factors (SMuRFs), namely smoking, diabetes mellitus, dyslipidaemia, and hypertension [2]. However, recent registries indicate a growing population of patients suffering an acute coronary syndrome (ACS) without any SMuRF (SMuRF-less patients) [3–6]. Over the past decade, the prevalence of such cases among patients presenting with ST-segment elevation myocardial infarction (STEMI) has increased from 13 to 27% in a large, national registry [3].

To date, scientific evidence on the pathogenesis and etiology of SMuRF-less AMI remains limited, although it constitutes an increasingly recognized clinical entity. A popular hypothesis implicates systematic inflammation and high levels of intra-coronary pro-inflammatory cytokines, but data are scarce. Pro-inflammatory and atherosclerosis biomarkers, such as lipoprotein (a) [Lp(a)], C-reactive protein and fibrinogen, have been shown to have higher specificity in predicting worse prognosis of AMI in SMuRF-less patients, as compared to patients with SMuRFs, but no studies have been conducted to investigate their role as predictors of SMuRF-less AMIs [4, 7]. Furthermore, large observational studies have indicated several demographic features potentially linked to SMuRF-less status in AMI, but the results are contradictory, probably due to marked heterogeneity in studied populations [8–10]. Hence, pinpointing sensitive clinical and laboratory parameters, as well as diagnostic approaches, for the prediction of AMI in patients without SMuRFs is crucial, as it concerns an increasing proportion of patients with CAD, who are rather under-represented in registries and clinical trials of cardiovascular risk-assessment [11].

The aim of this study is, therefore, to investigate potential clinical and/or laboratory characteristics associated with SMuRF-less AMIs by comparing the prevalence of clinical parameters and levels of laboratory and imaging indicators among patients with and without SMuRFs. The ultimate goal is the development of a predictive risk stratification model capable of recognizing patients without SMuRFs at high risk for AMI. Secondarily, the study aims at investigating differences in the prognosis of SMuRF-less patients compared to those with SMuRFs.

## Methods

### Study design and population

The ‘‘Beyond-SMuRFs Study’’ (ClinicalTrials.gov Identifier: NCT05535582) is an investigator-initiated, prospective, non-interventional cohort trial involving patients suffering from AMI and undergoing coronary angiography. The study is performed in accordance with the general principles outlined in the Declaration of Helsinki [12] and the rules of good clinical practice (GCP), and has been approved by the Ethics Committee of the Aristotle University of Thessaloniki (reference number: 136945/2022).

A total of 500 consecutive patients presenting with STEMI or Non-STEMI (NSTEMI) at two academic hospitals in Thessaloniki, Greece, undergoing primary or emergency coronary angiography, will be enrolled in the study. All eligible participants will provide informed written consent before enrollment. Patients with a history of previous AMI or previous coronary intervention, either percutaneous or surgical, will be excluded, as the calculation of the SYNTAX score for these patients is not possible. Detailed eligibility criteria are described in ​Table 1.

**Table 1:** Inclusion and exclusion criteria of the study population

Inclusion Criteria	Exclusion Criteria
• Age > 18 years • Hospitalization for acute myocardial infarction (AMI) with or without ST elevation (based on the Fourth Universal Definition of Myocardial Infarction) within the previous 4 weeks • Coronary angiography before or after hospitalization for AMI, in which at least one stenosis > 50% in a major epicardial coronary artery (left anterior descending artery, left circumflex artery, right coronary artery) or a branch thereof with a diameter of at least 2 mm was observed.	• Inability or refusal to provide informed consent • Age > 80 years • History of hospitalization due to AMI prior to the present AMI • History of coronary revascularization prior to the present AMI • Previous coronary angiography (prior to the present AMI) showing > 50% stenosis in a major epicardial coronary artery

Patients will be divided into two groups based on their medical history: (i) Group A: SMuRF-less patients, (ii) Group B: Patients with SMuRFs, defined as those who fulfilled at least one of the following criteria: (i) known history of hypertension and/or antihypertensive treatment prior to AMI, (ii) self-reported use of tobacco products on a systematic basis for up to 12 months before AMI, (iii) history of diabetes mellitus type 1 or 2 and/or treatment with antidiabetic tablets or insulin before AMI or diagnosis of diabetes mellitus based on HbA1c during AMI hospitalization, (iv) known hypercholesterolemia (total cholesterol > 200 mg/dl / LDLc > 150 mg/dl) or treatment with statins or PCSK9is, before AMI. SMuRF-less patients (Group A) are defined as those suffering an AMI in the absence of these comorbidities.

### Data collection and study procedures

After obtaining written informed consent, the following clinical characteristics will be recorded for each patient: demographics, socioeconomic parameters, complete medical history and medication, prior diagnostic and therapeutic interventions. A self-reported measurement of patients’ physical activity will be provided using the International Physical Activity Questionnaire (IPAQ), which will be completed by each patient before discharge. IPAQ is a validated questionnaire utilized to objectively measure and stratify physical activity by dividing populations into three levels: low, moderate and high physical activity [13]. Obesity will be diagnosed by categorization of body mass index (BMI; Kg/m2) measured before echocardiographic study. Recorded socioeconomic parameters will include education, marital status, employment, income, migration background and ethnicity. Moreover, the 36-item short form (SF-36) standardized questionnaire will be administered to obtain a self-reported measure of their health-related perception of quality of life before the AMI. In addition, patient laboratory data will be recorded on admission and during hospitalization. These include total blood count, standard biochemistry parameters, coagulation tests, thyroid hormone and thyroid-stimulating hormone levels, HbA1c, NTproBNP and HsTnT levels on admission, peak values of HsTnT, and NTproBNP. Moreover, levels of LP(a), apolipoproteins B and A1 (ApoB and ApoA1) interleukin-6 (IL-6) and soluble urokinase plasminogen activator receptor (suPAR) on admission will be assessed. Additionally, coronary angiographic images of every patient will be evaluated by two experienced independent interventional cardiologists blinded as to the demographic and clinical patient characteristics. Angiographic parameters such as lesion characteristics, coronary dominance and the SYNTAX score will be calculated.

A complete and comprehensive transthoracic echocardiographic assessment (TTE) will be performed within 24 hours from admission. All TTE studies will be conducted by certified sonographers/cardiologists using high-end scanners (e.g. Vivid E95, GE Healthcare, Chicago, IL, USA). All analyses will be separately performed by two dedicated expert cardiologists, blinded to the clinical data of all participants. All cardiac chamber sizing quantification, two-dimensional (2-D) and Doppler measurements will be performed in accordance with current recommendations [14, 15]. Simpson’s biplane method will be employed for the calculation of left ventricular ejection fraction (LVEF) for the left ventricle (LV) and abnormal values of conventional LV diastolic parameters will be determined based on recently published criteria [15]. LV diastolic function parameters include mitral inflow and annular velocities and the derived trans-mitral to averaged septal and lateral annular early diastolic velocity ratio (E/e’). All right ventricular (RV) systolic function parameters including tricuspid annular plane systolic excursion (TAPSE), fractional area change (FAC), systolic movement of the RV lateral wall using tissue Doppler imaging (S’), pulmonary artery systolic pressure (PASP) will be evaluated as per current guidelines [16].

Two-dimensional (2D) speckle tracking echocardiography will be employed to calculate strain measurements for the LV, left atrium (LA), and RV. Global longitudinal strain (GLS) will be derived from the calculation of the average of the peak systolic longitudinal strain of all segments for each chamber. To estimate myocardial work indices, LV GLS and peak systolic LV pressure measurements will be integrated to the aforementioned module [17]. Four different indices of myocardial work will be calculated including (i) LV global work index (LVGWI, mmHg %), representing the total work within the LV pressure-strain loops, (ii) LV global constructive work (LVGCW, mmHg %), defined as the work performed during myocardial shortening in systole and the work during myocardial lengthening in isovolumic relaxation, (iii) LV global wasted work (LVGWW, mmHg %), representing the work contributing to the lengthening of the cardiac myocytes during systole and the shortening during isovolumic relaxation, and (iv) LV global work efficiency (LVGWE, %), defining the percentage of effectively spend work by the LV myocytes and obtained by the following formula: (LVGCW/[LVGCW + LVGWW]) × 100%.

**Fig. 1:**
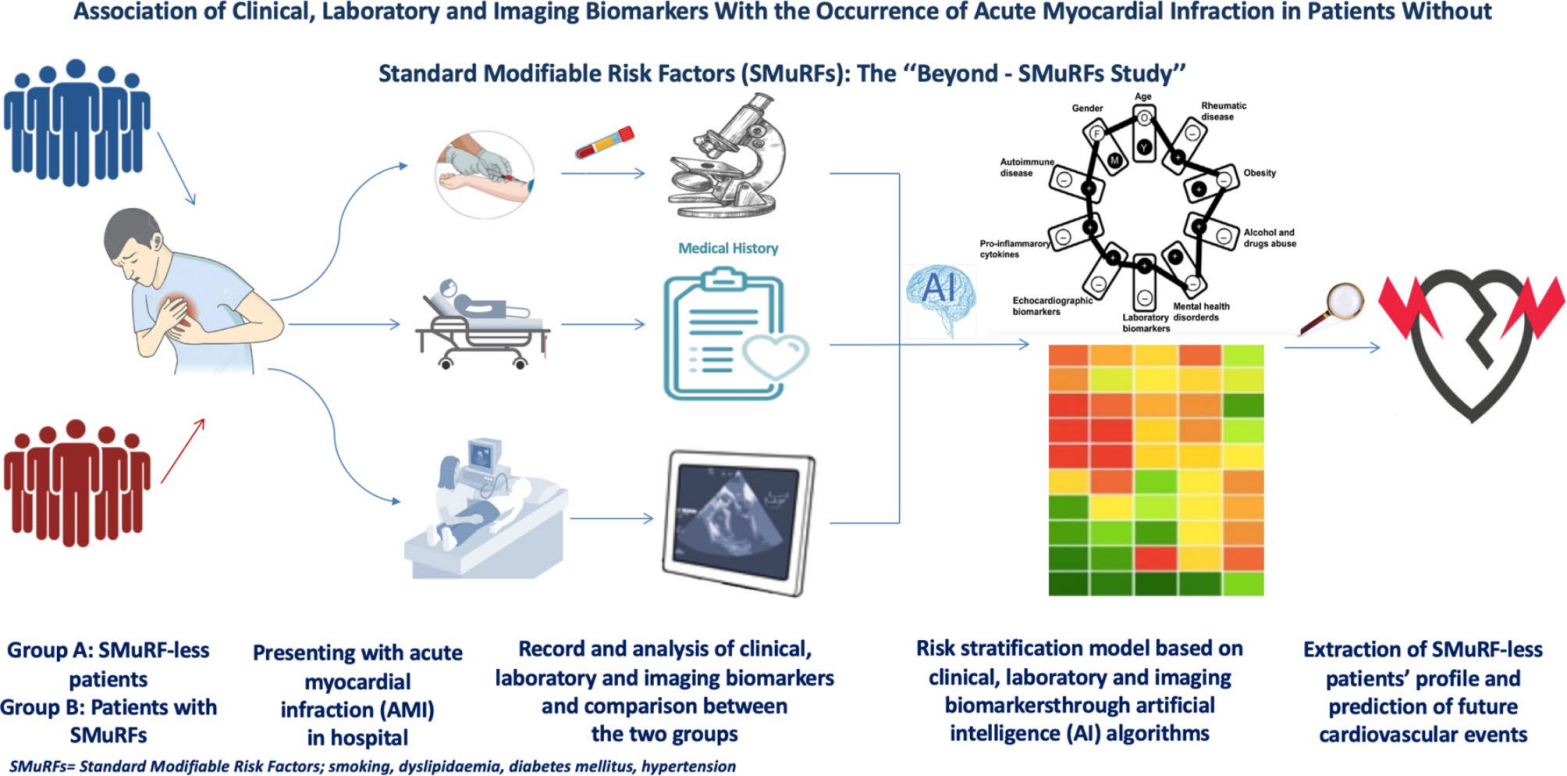
Visual Overview of the ‘‘Beyond-SMuRFs Study’’. Study processes required for the association of SMuRF-less myocardial infarctions with patients’ clinical, laboratory and imaging biomarkers (primary outcome) are depicted. (* This figure is original and, therefore, permission for publication was not needed to be obtained from a third-party)

The primary outcome of the study is to compare clinical, laboratory and imaging parameters among SMuRF-less patients and patients with SMuRFs, thereby exploring clinical, laboratory, echocardiographic and angiographic biomarkers potentially associated with SMuRF-less status in AMI. Subsequently, we aim to assess these parameters as potential independent predictors of SMuRF-less AMIs (logistic regression analysis). Secondary goals include a comparison of short- and long-term mortality and major adverse cardiovascular events (MACE), as well as of the complexity and severity of CAD, between AMI patients with and without history of SMuRFs (Fig. 1). Short-term outcomes include all-cause death and MACE during hospitalization and/or 30 days after hospital admission. Patients will also be followed-up for a median period of 24 months after enrollment to evaluate long-term prognosis. All deaths will be ascertained by searching in the Greek web-based national health insurance system. Apart from death, MACE will be documented by independent physicians either through hospital reports or via in-person or telephonic interviews. The first patient was enrolled in April 2022 and completion of enrolment is expected until August 2023.

### Statistical analysis

Clinical parametersc laboratory findings and imaging indices of interest will be compared among patients with and without SMuRFs to phenotypically characterize the SMuRF-less group, and identify any particular associations of these biomarkers with the SMuRF-less status. Subsequently, univariate logistic regression analysis will be performed to identify independent predictors of SMuRF-less AMIs. A multivariate logistic regression model will be constructed by forcing univariably significant and clinically relevant variables into the multivariable model. The G*Power software was utilized by a specialist statistician in order to calculate the sample size required to derive statistical significance. It was estimated that to detect an odds ratio > 2 with a prevalence of 15% of SMuRF-less status among AMIs, approximately 75 SMuRF-less patients will be required (with a probability for Type I error: 0.80, and statistical significance level: 0.05). Therefore, 500 patients (with and without SMuRFs) will be included in the present study. An odds ratio > 2 will be interpreted as a prediction of a 2-fold higher probability of having SMuRF-less AMI than an AMI attributed to SMuRFs.

Baseline patient characteristics of each group will be examined and compared using the chi-square test for categorical variables and the 2-sided Student’s t-test for continuous variables or non-parametric tests (Wilcoxon, Mann-Whitney U etc.), when assumptions of normality are not met. Categorical variables will be represented by frequencies and percentages (%) and continuous variables will be summarized by mean ± standard deviation (SD) or median (1st -3rd quartile), as appropriate. In terms of statistical tests recruited for outcomes calculation, the two groups of patients will be compared to each other to identify clinical, laboratory and imaging biomarkers associated with a higher or lower probability of developing a SMuRF-less AMI. The chi-square test will be used to compare the prevalence of clinical parameters among patients with and without SMuRFs, and the t-test and Mann-Whitney U test will be utilized to compare the mean levels of laboratory and imaging biomarkers between the two groups. As mentioned before, univariate linear and logistic regression analyses will be applied to calculate univariate odds ratios, and then multivariate regression analyses will be performed. Finally, in order to identify the optimal combination of epidemiological, clinical, laboratory and imaging biomarkers associated with SMuRF-less AMIs, supervised machine learning algorithms will be used. The resulting clinical-laboratory prediction models will be evaluated with Receiver Operating Characteristic (ROC) curves.

A time-to-event analysis will be performed to assess whether the absence of SMuRFs is associated with better or worse clinical prognosis of patients (secondary outcome). Event rates will be compared by the long-rank test. Μultivariable Cox proportional hazard models will be utilized to adjust the results for clinically relevant and univariately significant variables. The 2-tailed p value of 0.05 will be considered the significance threshold for all statistical tests. All outcomes will be reported with 95% confidence intervals. Data management and statistical analyses will be conducted using SPSS software, version 26 (IBM SPSS Statistics) and R version 3.4.4 (R Foundation for Statistical Computing, Vienna, Austria).

## Discussion

‘’Beyond-SMuRF’’ is a prospective cohort trial, enrolling recently hospitalized patients with AMI and aspiring to identify clinical, laboratory and imaging biomarkers associated with SMuRF-less status. So far, suggested polygenic clinical risk-score models seem to underestimate the risk in SMuRF-less patients and are usually incapable of identifying cardiovascular risk factors on top of SMuRFs [18]. Therefore, the rationale of this study is -by comparing clinical, laboratory, echocardiographic and angiographic parameters between the SMuRF-less group and the group of patients with SMuRFs- to assess novel biomarkers as predictors of SMuRF-less AMIs and ultimately generate a risk-stratification tool for this increasingly recognized entity. To our knowledge, this is the first study to comprehensively attempt an in-depth, multiparametric evaluation of the profile of SMuRF-less population with AMI. Although several studies have reported clinical outcomes in SMuRF-less patients, there is a lack of evidence regarding clinical, laboratory and imaging findings in these patients.

Several studies have reported baseline demographic characteristics among patients with and without SMuRFs [3, 11, 18, 19]. Ηowever, no marked differences have been explored so far between the two groups in terms of basic demographic features such as age and sex. Moreover, differences in baseline clinical characteristics and comorbidities have not been thoroughly evaluated on a systematic basis, which adds to the novelty of this study. For instance, rheumatic and autoimmune diseases, as well as abdominal obesity, alcohol consumption and drug use, have been associated with increased cardiovascular risk but have never been assessed as potential absolute explainers of SMuRF-less AMIs [18, 20]. Furthermore, mental health status and psychosocial risk factors have been linked to CAD, but data regarding the association thereof with SMuRF-less status in AMI are lacking [20–24]. Additionally, the lack of physical activity, as well as specific socioeconomic parameters, have been proven drivers of CAD, but never evaluated in the context of SMuRF-less AMIs [24–26].

Moreover, the laboratory profile of SMuRF-less patients remains understudied. In general, there is an absence of available and specific blood work-up biomarkers of CAD beyond markers indicating SMuRFs. In a retrospective analysis of the SWEDEHEART registry, the authors found lower body-mass index, lower triglyceride concentrations, and higher HDL-C concentrations in the SMuRF-less group versus patients with SMuRFs, which probably suggests that these factors might not drive the atherosclerosis [27]. Only few studies have been conducted to indicate blood markers associated with AMI in these patients, but none of them showed remarkable results or studied non-conventional biomarkers [4, 27, 28]. Consistent with the hypothesis that a significant number of mechanisms underlying atherosclerosis and ACS without SMuRFs remains undiscovered, our study will be the first to elaborate metabolomic and inflammatory biomarkers, such as Lp(a), ApoB and ApoA1 interleukin-6 (IL-6) and suPAR. Although an increasing number of registries assess the impact of such biomarkers on patients with AMI, none of them have focused on SMuRF-less patients [29–31]. The logic behind this effort is that the metabolic and inflammatory status of patients have been shown to play a pivotal role in cardiovascular disease and specifically in CAD and ACS [32–40]. On top of that, genetic analyses, Mendelian randomization studies, and the determination of specific polymorphism responsible for atherogenesis in SMuRF-less patients should drive future research to elucidate additional pathogenetic aspects, and develop -potentially with the use of artificial intelligence- clinically relevant polygenic risk scores [41, 42].

Regarding imaging biomarkers, the innovation of the study lies in the fact that it recruits conventional and novel echocardiographic parameters and analytical angiographic assessment to unravel differences among SMuRF-less patients compared to patients with SMuRFs. As novel imaging modalities have proved, plaque vulnerability and rupture electromechanical complications are not always directly associated with systematically assessed biomarkers and risk factors such as dyslipidaemia [43, 44]. Moreover, an observational study by Figtree et al. indicated differences in the prevalence of left main and left anterior descending culprit lesions in SMuRF-less patients compared to those with SMuRFs [27]. As explained by the authors, this may be partly attributed to the family history of premature CAD and is an important finding potentially leading to a more adverse AMI risk profile in SMuRF-less patients. A potential criticism that our study could deal with is that differences in imaging parameters between patients with and without SMuRFs are likely to be the consequence of AMI rather than a predictive parameter. However, the main objective of the study is to identify associations of clinical, laboratory and imaging variables with SMuRF-less status which should be tested in future larger trials for their predictive value. Thus, the elaboration of additional biomarkers, such as imaging parameters, could contribute to the characterization of the clinical profile of SMuRF-less patients hospitalized with AMI, and shed light on the etiology of this emerging clinical entity. Finally, this study aims to provide data on the prognosis of SMuRF-less AMIs and evaluate emerging evidence highlighting the worse clinical course of these patients [3, 5, 6, 45–49]. The reason behind this observation could be correlated with increased time-to-reperfusion time due to reduced suspicion of AMI in these individuals, but also with undiscovered pathogenetic mechanisms and ‘‘hidden’’ comorbidities which might increase their cardiovascular risk.

### Limitations

Certain limitations of this study should be properly acknowledged. The main limitation is its observational nature which does not allow to conclude causal associations. Second, this study is not a multi-center one and its population consists of Greek patients exclusively. Future studies should be conducted to include and study other ethnicities and races to account for inherent variability of different patient populations and test the generalizability of our results. Moreover, the authors set an upper age limit as inclusion criterion in the study in an attempt to exclude very old patients with age-relating comorbidities and intricated AMI pathophysiology. As there are no evidence on cutoff values to support this limit, this could lead to selection bias in the study and ambiguous resuls across different age groups. Finally, our study -due to restricted resources- will study only some of the potential predictive laboratory biomarkers. Comprehensive and complete analysis of SMuRF-less patients’ metabolomic and genetic profiling should be taken into consideration for future research.

This real-world, prospective, non-interventional cohort trial study of patients hospitalized with AMI has the potential to identify clinical, laboratory and imaging biomarkers associated with the occurrence of AMI in SMuRF-less patients. SMuRF-less individual profiling could ultimately lead to the development of personalized risk-stratification models predicting adverse cardiovascular events.

**and acronyms**:

ACS = acute myocardial infarction; CAD = coronary artery disease; NSTEMI = Non-ST-segment elevation myocardial infarction; SmuRF = standard modifiable risk factors; STEMI = ST-segment elevation myocardial infarction.

## Data Availability

The datasets used and/or analysed during the current study will be available from the corresponding author on reasonable request.
