# Correction: Association of clinical, laboratory and imaging biomarkers with the occurrence of acute myocardial infarction in patients without standard modifiable risk factors – rationale and design of the “Beyond-SMuRFs Study”

**DOI:** 10.1186/s12872-023-03243-6

**Published:** 2023-04-25

**Authors:** Dimitrios V. Moysidis, Stylianos Daios, Vasileios Anastasiou, Alexandros C. Liatsos, Andreas S. Papazoglou, Efstratios Karagiannidis, Vasileios Kamperidis, Kali Makedou, Aikaterini Thisiadou, Paraskevi Karalazou, Marios Papadakis, Christos Savopoulos, Antonios Ziakas, George Giannakoulas, Vassilios Vassilikos, Georgios Giannopoulos

**Affiliations:** 1 Third Department of Cardiology, Hippokration General Hospital, School of Medicine, Faculty of Health Sciences, Aristotle University of Thessaloniki, Konstantinoupoleos 49, 54642 Thessaloniki, Greece; 2 First Department of Cardiology, School of Medicine, Faculty of Health Sciences, AHEPA University Hospital, Aristotle University of Thessaloniki, St. Kyriakidi 1, 54636 Thessaloniki, Greece; 3 Athens Naval Hospital, Dinokratous 70, 11521 Athens, Greece; 4 Laboratory of Biochemistry, Faculty of Health Sciences, School of Medicine, AHEPA General Hospital, Aristotle University of Thessaloniki, St. Kyriakidi 1, 54636 Thessaloniki, Greece; 5 University Hospital Witten-Herdecke, University of Witten-Herdecke, Heusnerstrasse 40, 42283 Wuppertal, Germany; 6 First Propedeutic Department of Internal Medicine, Aristotle University of Thessaloniki, AHEPA University Hospital of Thessaloniki, Thessaloniki, Greece

**Correction: BMC Cardiovasc Disord 23, 149 (2023)**

**https://doi.org/10.1186/s12872-023-03180-4**

Following publication of the original article [1], the surname and the given name need to be replaced for the authors Aikaterini Thisiadou, Paraskevi Karalazou in the author group.

The original article has been corrected.

## References

[1] Moysidis DV (2023). Association of clinical, laboratory and imaging biomarkers with the occurrence of acute myocardial infarction in patients without standard modifiable risk factors – rationale and design of the “Beyond-SMuRFs Study”. BMC Cardiovasc Disord.

